# Effects of M-DEPTH Model of Depression Care on Maternal HIV Viral Suppression and Adherence to the PMTCT Care Continuum Among HIV-Infected Pregnant Women in Uganda: Results from a Cluster Randomized Controlled Trial at Pregnancy Completion

**DOI:** 10.1007/s10461-023-04014-2

**Published:** 2023-03-13

**Authors:** Glenn J. Wagner, Violet Gwokyalya, Laura Faherty, Dickens Akena, Janet Nakigudde, Victoria Ngo, Ryan McBain, Bonnie Ghosh-Dastidar, Jolly Beyeza-Kashesya, Juliet Nakku, Leticia Kyohangirwe, Linda Kisaakye Nabitaka, Hafsa Lukwata, Barbara Mukasa, Rhoda K. Wanyenze

**Affiliations:** 1 RAND Corporation, 1776 Main Street, Santa Monica, CA 90407 USA; 2 Makerere University, School of Public Health, Kampala, Uganda; 3 Boston University School of Medicine, Boston, MA USA; 4 College of Health Sciences, Makerere University, Kampala, Uganda; 5 City University of New York, Graduate School of Public Health and Health Policy, New York, USA; 6 Mulago Specialized Women and Neonatal Hospital, Kampala, Uganda; 7 Makerere University, School of Medicine, Kampala, Uganda; 8 Butabika National Referral Mental Hospital, Kampala, Uganda; 9 Ministry of Health, Kampala, Uganda; 10 Mildmay Uganda, Kampala, Uganda

**Keywords:** Perinatal depression, HIV, Uganda, Problem solving therapy, antidepressants, PMTCT, Adherence, Viral suppression

## Abstract

Perinatal depression has been shown to impede adherence to antiretroviral therapy (ART) and the prevention of mother-to-child transmission (PMTCT) care continuum; therefore, treating perinatal depression may result in increased viral suppression and PMTCT adherence. We examined the effects of the M-DEPTH (Maternal Depression Treatment in HIV) depression care model (including antidepressants and individual Problem Solving Therapy) on depression, maternal viral suppression and adherence to PMTCT care processes in an ongoing cluster-randomized controlled trial of 391 HIV-infected pregnant women (200 usual care; 191 intervention) with at least mild depressive symptoms enrolled across 8 antenatal care clinics in Uganda. At baseline, 68.3% had clinical depression and 41.7% had detectable HIV viral load. Adjusted repeated-measures multivariable regression models found that the intervention group was nearly 80% less likely to be clinically depressed [Adjusted OR (95% CI) 0.22 (0.05, 0.89)] at the 2-month post-pregnancy assessment, compared to the control group. However, the intervention and control groups did not differ meaningfully on maternal viral suppression, ART adherence, and other PMTCT care processes and outcomes. In this sample of women who were mostly virally suppressed and ART adherent at baseline, the depression care model had a strong effect on depression alleviation, but no downstream effects on viral suppression or other PMTCT care processes.

*Trial Registration* NIH Clinical Trial Registry NCT03892915 (clinicaltrials.gov).

## Introduction

Prevention of mother-to-child transmission (PMTCT) care has been scaled up across Uganda and the larger region of Sub-Saharan Africa (SSA) in efforts to eliminate vertical transmission of the HIV virus from the a mother living with HIV to her fetus or infant [1]. Nonetheless, one in five new HIV cases in Uganda still result from mother-to-child transmission [2]. Despite recent evidence suggesting that nearly all Ugandan pregnant women living with HIV receive PMTCT care and are on ART [2], about one in three do not adhere to the full PMTCT care continuum, which includes maternal pre- and post-natal use of antiretroviral therapy (ART), infant use of antiretroviral prophylaxis, periodic infant HIV testing, and exclusive breast-feeding [3, 4].

Depression is common among pregnant and post-partum women living with HIV, and depression may contribute to poor adherence to PMTCT care processes. Studies in SSA suggest 30–50% of women living with HIV suffer from elevated depressive symptoms either during pregnancy or post-partum [5], with higher rates in women who are newly diagnosed with HIV [6], and over one-third are estimated to be clinically depressed and in need of treatment [5, 7]. In pregnant women, depression is related to more rapid HIV disease progression and mortality [8], as well as poor use of and adherence to ART [9]. One recent study found perinatal depression doubled the likelihood of poor ART adherence [10]. Furthermore, perinatal depression has harmful effects on birth outcomes and early child development [11, 12].

**Fig. 1:**
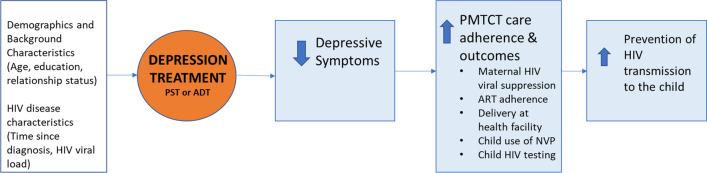
Conceptual framework for the effects of depression treatment on depression, PMTCT care processes, and HIV transmission to the child

Depression treatment and depression alleviation have been shown to improve adherence to HIV care processes [13–16], but such benefits have not been evaluated in the more complex PMTCT care continuum. We hypothesize that use of evidence-based treatment of perinatal depression would result in reduced depressive symptoms, which in turn would improve adherence to ART, care retention and other PMTCT care processes, and this would contribute to maternal HIV viral suppression and reduced risk of vertical HIV transmission (see Fig. 1).

Depression is rarely diagnosed and treated in Ugandan antenatal care (ANC) or HIV clinics, in part due to the scarcity of mental health professionals [17]. Collaborative depression care models have used various forms of task-shifting to overcome human resource constraints and successfully deliver evidence-based depression care in low resource settings [18–20], including HIV clinics in Uganda [21]. Studies in SSA have shown that with training and supervision from mental health specialists, lay persons and nurses are able to effectively screen for and diagnose depression, as well as administer evidence-based depression care, including forms of talk therapy such as Problem Solving Therapy [22] and antidepressants [21, 23]. Similar depression care models have been effective for perinatal depression in the U.S. [24, 25], but have not been studied with this population in SSA or with PMTCT care.

Maternal Depression Treatment in HIV (M-DEPTH) is an ongoing cluster randomized controlled trial of evidence-based depression treatment to alleviate depression and ultimately improve PMTCT care adherence and outcomes among women living with HIV in Uganda who are experiencing perinatal depression [26]. The primary objective of the study is to assess whether the integrated depression care model is superior to usual care in helping women to adhere to each step of the PMTCT care continuum, via depression alleviation. All participants have completed their pregnancy, enabling us to examine the effects of the intervention on depression as well as the primary outcome of maternal viral suppression at pregnancy completion, and other care processes that take place prior to and/or shortly after the woman has given birth to her infant.

## Methods

### Study Design

This is a prospective, multi-site cluster randomized controlled trial to evaluate the effects of an evidence-based, collaborative care model of depression care, relative to usual care, on PMTCT adherence and outcomes. The study is being performed at eight ANC clinics in Uganda, with four clinics randomly assigned (using a computer-generated list of assignments) to implement the intervention in addition to usual care, and four clinics to implement usual care alone. Randomization was stratified by level of facility (larger regional hospital and smaller health center IVs), which differ primarily by size of clientele, as the ANC and mental health services are comparable across all sites, as is staff composition. To ensure balance on this factor, clinics were randomized such that one regional hospital and three health center IVs (HCIV) were assigned to each of the intervention and control arms. Providers, participants, and data collectors were unblinded to the assignment of treatment condition; only the data analyst was blind. Participants were followed through completion of pregnancy and until 18-month post-partum (six months for women who had a miscarriage, abortion, or still-birth), with assessments at baseline and 2, 6, 12 and 18 months post pregnancy completion; however, only data from the baseline and 2-month post-pregnancy completion (PP) assessments have been fully collected and thus included in this analysis. The study protocol was reviewed and approved by institutional review boards at RAND and Makerere University School of Public Health, and the Uganda National Council for Science and Technology.

### Study Setting

The participating ANC clinics were located within public health facilities operated by the Uganda Ministry of Health, and each received technical assistance in HIV reproductive health care from Mildmay Uganda. Each of the participating health facilities include an HIV clinic, a maternal and child health center (which houses the ANC clinic as well as post-partum Mother-Baby Care clinic for mothers living with HIV), in-patient pediatric ward, and other services. When a client of the HIV clinic becomes pregnant, she is transferred to the ANC clinic to receive PMTCT and pregnancy care, including ART; if she first learns of her HIV diagnosis when entering ANC, then she remains there. She receives care within the maternal and child health center until her newborn is 18 months old, or her pregnancy is prematurely terminated, after which she returns to the HIV clinic for continued care. Each ANC clinic is staffed by one doctor-in-charge (who plays more of a supervisory role), two to three midwife nurses (who prescribe treatment), and one or two peer mothers (former clients living with HIV who successfully managed their pregnancy and who provide volunteer assistance). The hospital ANC clinics service 400–600 women living with HIV annually. Women typically enter ANC care in the second trimester and are seen monthly during antenatal and early postpartum phases. Delivery takes place in the in-patient maternity ward.

### Participants

Recruitment took place between July 2019 and January 2021. Women attending the ANC clinic were eligible for the study if the following criteria were met: (1) gestation period was 32 weeks or less, (2) HIV-positive (confirmed by medical provider), (3) age 18 years or older, and (4) scored > 4 on the 9-item Patient Health Questionnaire (PHQ-9) [27] as an indication of at least some depressive symptoms. Women were excluded if they were currently receiving mental health treatment. At each site, all adult clients living with HIV who were early enough in their gestation period to be eligible for the study were screened for potential depression by trained peer mothers using two items (loss of interest, depressed mood) from the PHQ-9 (PHQ-2) at routine clinic visits. Those who screened positive on the PHQ-2 (i.e., a score greater than zero) received depression psychoeducation and were informed of the study; this cutoff (> 0), rather than the conventional ≥ 3 cutoff, is supported by other research with this screening instrument in SSA [28]. Eligible women who expressed interest in participating in the study were further evaluated by a nurse who administered the full 9-item PHQ-9. Women who scored > 4 on the PHQ-9, were medically stable (i.e., no acute opportunistic infections and were on ART for at least 4 weeks), and not displaying a high risk for suicide, were then referred to the study coordinator to have their eligibility confirmed and receive informed consent procedures. All women who enrolled provided written informed consent.

Our target sample size was 400 participants (50 per site), which was calculated to provide sufficient power to detect a 7-percentage point difference between the two study arms (Cohen’s δ = 0.16) for undetectable viral load at PP, assuming an attrition rate of 10%, intracluster correlation coefficient estimate of 0.01 [29], and 70% of the control group achieving undetectable viral load [30].

### Treatment Conditions

#### Usual Care

Usual care procedures for addressing depression in the participating ANC clinics (and other public ANC clinics across the country) are limited to referral of patients exhibiting severe depressive symptoms to a mental health specialist (typically a psychiatric nurse) either at the facility or at the District or Regional Referral Hospital. In addition, each study site (like most public ANC clinics in Uganda) offers Family Support Groups (FSG) to clients living with HIV to provide psychosocial support, instruction and education to support prenatal and post-partum care, including PMTCT adherence [31]. The manualized FSG curriculum is comprised of 24 monthly group sessions from the antenatal phase through 18 months post-partum; each session is two hours and led by the peer mothers and nurses on staff.

#### M-DEPTH Depression Care Model

Drawing from evidence-based collaborative care models for depression in low resource settings [18, 19], we used a stepped care approach to offering psychological and pharmacologic treatment options. The primary components of the depression care model consisted of (1) *depression screening* implemented by peer mothers using the PHQ-2 for adult pregnant women living with HIV attending routine ANC visits; (2) *evaluation of depressive disorder and treatment eligibility,* which consisted of the PHQ-9 screen by the nurse, and for those who scored > 9: the application of the Mini International Neuropsychiatric Interview (MINI)^32^ criteria for major depression, including functional impairment and psychiatric rule outs (high suicide risk, psychosis, mania, substance abuse disorder), and assessment of medical stability (no acute, untreated infections; on ART for at least 4 weeks); (3) *depression psychoeducation* and recommendation of treatment modality by the nurse [problem solving therapy (PST) for moderate depression (PHQ-9 scores of 10–19) and antidepressant therapy (ADT) for severe depression (PHQ-9 > 19), consistent with WHO mhGAP guidelines [32]; and (4) *provision of treatment* (PST or ADT) as selected by the client. Participants with PHQ-9 scores between 5 and 9 received psychoeducation and continued depression monitoring at monthly usual care visits. A Depression Care Registry was maintained by the nurses and peer mothers to record treatment data for each visit, which facilitated treatment monitoring at future visits, supervision, and fidelity assessment. Peer mothers and midwife nurses on staff at the clinics were trained during a 3-day workshop. Supervision from mental health specialists hired by the study was held weekly for the first two months, then biweekly for four months, and then monthly thereafter. To monitor fidelity, supervisors reviewed client charts and the Depression Care Registry at each supervision. A more detailed description of the model is available in a prior publication [26].

##### Problem Solving Therapy (PST)

PST is a cognitive-behavioral intervention that trains recipients on adaptive problem-solving attitudes and the deliberate and systematic application of four problem-solving skills: problem definition, generation of possible solutions, selection of solutions to use, and implementation and evaluation of solutions [33–35]. Peer mothers were trained to implement manualized individual therapy that consisted of three biweekly core sessions to orient the client to PST principles and methods, followed by up to four ancillary monthly sessions as needed (maximum of seven total sessions) for those continuing to experience depressive symptoms; therefore, therapy was typically completed in four to five months.

##### Antidepressant Therapy (ADT)

ADT was administered and monitored by nurses, and was not prescribed until after the first trimester to reduce teratogenicity risks to the mother’s infant [36]. Fluoxetine was the medication typically prescribed because it is well tolerated and has been extensively researched in the perinatal context [37, 38], although imipramine was also available. The starting daily dose of fluoxetine was 20 mg, and 50 mg for imipramine (increasing to 75 mg after the first week). Follow-up visits were monthly, at which time dose increments or medication changes were considered based on measures of treatment response and side effects; in the post-partum phase, mothers were instructed to monitor their infant for symptoms of poor neonatal adaptation (e.g., excessive crying, jitteriness). Discontinuation was considered when symptoms were in remission (PHQ-9 < 5) for 6 months [39], and if the mother was at least 6 months post-partum, given the heightened risk of depression post-partum and in the event that she had a miscarriage or abortion.

### Measures

Assessments included a survey, laboratory assays, pharmacy data, and data abstracted from medical charts and the Depression Care Registry. Survey measures that had not been translated into Luganda during our prior research were translated using standard translation, back-translation methods, and were interviewer-administered.

#### Adherence to PMTCT Care Continuum

These measures included the following: *ART use and adherence* [percent of prescribed pills taken: (pills dispensed/pills prescribed per day)/days between refills, multiplied by 100] was determined using pharmacy refill data for the time between baseline and PP. *Retention in ANC/HIV care* was assessed through clinic attendance abstracted from medical charts. Participants were considered to be retained in care if they had been seen at the ANC clinic at least once in the three months prior to completion of pregnancy, and were considered still in care by the clinic as of PP. *Infant antiretroviral use* [i.e., nevirapine (NVP)] during the first 6 weeks of life was assessed by chart abstraction and maternal self-report. *Infant HIV testing* (at week 6) and *serostatus* was ascertained via chart abstraction. *Location of infant delivery* (i.e., whether the delivery took place at a health care facility) for participants who carried their pregnancy to term, was assessed via chart abstraction and participant self-report.

#### Maternal Virologic Suppression

HIV viral load tests were performed by the study at enrollment and the PP assessment. Blood samples were transported to a central lab (Uganda Virus Research Institute) for processing using the Roche Tagman assay, which is a real-time reverse transcriptase PCR. The primary analysis used a binary variable representing undetectable (< 20 copies/mm^3^) viral load at the PP assessment; log_10_ viral load change from baseline to PP was used in secondary analysis.

*Pregnancy outcome* (live birth, miscarriage/abortion, still birth, neonatal or maternal death) was ascertained through medical chart abstraction and maternal self-report.

#### Depression

The 9-item Patient Health Questionnaire (PHQ-9) [27] was used to assess depression. Each item corresponds to the nine symptoms assessed in the depression module of the Diagnostic Statistical Manual of Mental Disorders [40]. Each item is scored from 0 to 3 to represent frequency of symptom presence in the past two weeks, and scores > 9 (possible range: 0 to 27) represent clinical depression and have been shown to correspond highly with Major Depression diagnosis [27]. The PHQ-9 has been used successfully to assess depression in sub-Saharan Africa [41], including among pregnant women living with HIV [42]. A recent systematic review and meta-analysis of the use of the PHQ-9 in the perinatal context, revealed strong psychometric properties for the scale in comparison to standard psychiatric interview and the Edinburgh Postnatal Depression Scale [43].

*Receipt of mental health services* were assessed at the PP assessment by asking participants if they had received any formal psychotherapy or medication (e.g., antidepressants) for mental health problems since enrollment in the study.

*Demographics* measured included age, education level (binary indicator of any formal secondary education), and relationship status (binary indicator of being in a committed relationship).

*HIV disease characteristics* measured included time since HIV diagnosis, time in HIV care, and time on ART.

### Data Analysis

We conducted bivariate analyses to compare the study arms on baseline measures of participant characteristics (i.e., demographics, HIV disease, stage of pregnancy, depression). We assessed differences using chi-square tests for categorical variables, and two-tailed independent t-tests for continuous variables. We repeated this analysis to compare those who completed the PP assessment with those who did not.

The primary analyses followed an intent-to-treat (ITT) approach; for outcomes with missing data at PP, the baseline measure of the outcome was used, and if a baseline measure was not available (e.g., pregnancy outcome), the participant was assigned the negative outcome (e.g., pregnancy did not result in a successful delivery). We chose not to use weights or imputation, given the low level of item nonresponse at the PP assessment. The primary objective of the analysis was to examine the effects of the M-DEPTH depression care intervention on maternal viral suppression at PP; the primary outcome was undetectable viral load, and the secondary outcome was amount of log_10_ viral reduction from baseline to PP. We first conducted chi-square or independent 2-tailed t-tests to assess associations between the outcome and treatment condition. We estimated logistic regression for binary outcomes, and linear regression for continuous outcomes, with adjustments for the following covariates: baseline demographics (age, any secondary education, relationship status), weeks on ART by PP, weeks between baseline and PP, number of FSG sessions attended in the six months prior to PP, and presence of undetectable HIV viral load at baseline (except for models of the secondary outcome, which used log_10_ viral load at baseline). We elected not to include a covariate related to receipt of, or level of exposure to, depression treatment, because nearly all intervention participants who were deemed eligible for treatment (PHQ-9 > 9) received at least 8 weeks of treatment, which is an adequate dose to evaluate the effects of treatment.

To assess the effects of the intervention on intermediary outcomes that our conceptual model (see Fig. 1) posits are the pathways by which the intervention affects maternal viral suppression, we used the same analytic approach (and covariates) to conduct logistic regression models that examined intervention effects on depression status at PP, and good (100% of prescribed doses taken) ART adherence between baseline and PP. Further, to examine the posit of our conceptual model that alleviated depression improves ART adherence, we used the analytic approach described above to assess the association between depression status at PP, and reduction in depressive symptoms from baseline to PP, on good ART adherence between baseline and PP, in separate analyses. In these models, an indicator for treatment condition was not included, while depression at baseline (depression status for the model that included depression status at PP; PHQ-9 score for the model that included reduction in depressive symptoms) was added as a covariate.

In a secondary analysis to assess whether the intervention may have greater effects on women who were clinically depressed and in need of depression treatment at baseline, we replicated all the analyses described above with the subgroup of participants who had clinical depression (PHQ-9 > 9) during the enrollment screening. We also replicated these analyses with data only from the participants who completed the PP assessment; the findings from this sensitivity analysis were essentially equivalent to the ITT analysis and thus not reported in this paper.

We used only bivariate regression models to examine intervention effects on outcomes that were highly positively skewed in both study arms (i.e., pregnancy outcome, location of delivery, child HIV testing and status, child use of NVP and cotrimoxazole), and resulted in no group differences.

All group comparisons included an adjustment for clustering within site. For estimation, we used Proc Surveylogistic or Proc Surveyreg in SAS 9.2 to allow for clustering.

## Results

### Sample Characteristics

**Fig. 2:**
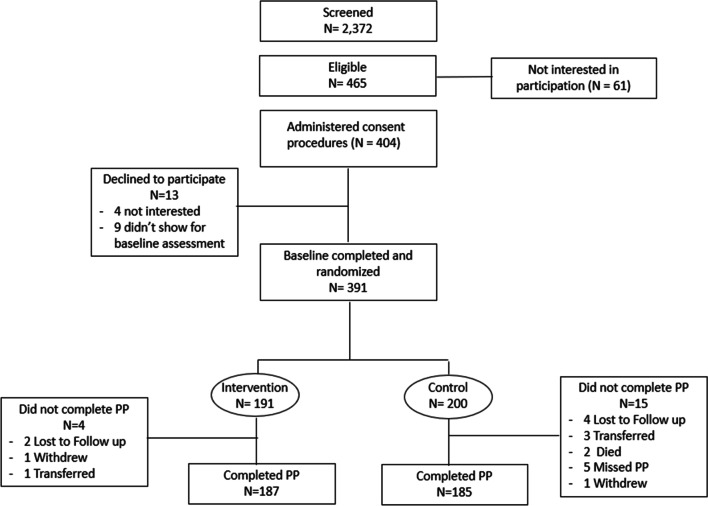
Flow of participants from screening and enrollment to the two-month post-pregnancy (PP) assessment

A total of 2372 adult HIV-infected pregnant women with no more than 32 weeks of gestation were screened for study eligibility across the eight study sites; 694 (29.3%) screened positive on the PHQ-2, of whom 627 (90.3%) expressed interest in study participation and were further evaluated for eligibility. Of these 627, 586 (93.5%) were medically stable and therefore administered the PHQ-9; 465 of the 586 women (79.4%) scored > 4 on the PHQ-9 and were eligible, of whom 404 expressed interest in study participation and were referred to the study coordinator for consent procedures. Of these 404, 391 (96.8%) agreed to enroll in the study and comprised the analytic sample (see Fig. 2), while the remaining 13 decided they were not interested (4) or did not return for the baseline assessment (9).

Of the 391 enrolled women, 50 were enrolled at each of 7 of the 8 study sites, while the eighth site enrolled 41 (200 at control sites; 191 at intervention sites). Table 1 lists the baseline characteristics of the participants. Women in the two study arms did not differ on demographics, nor stages of HIV disease or gestation (see Table 1). Study retention was high, with 372 (95.1%) women completing the PP assessment; of the remaining 19 (15 control, 4 intervention), 11 were lost to follow-up or missed the assessment, four moved and transferred their care, two died after giving birth, and two withdrew from the study shortly after enrollment. Those who completed the PP assessment were older (mean age = 27.6 vs. 24.8; t = 3.8, df = 7, p = 0.045), more likely to be in a committed relationship (83.3% vs. 63.2%; t = 3.7, df = 7, p = 0.02), and had been diagnosed with HIV (mean months = 46.1 vs. 25.6; t = 3.2, df = 7, p = 0.046) and on ART (mean weeks = 176 vs. 85; t = 5.1, df = 7, p = 0.001) for longer periods of time compared to those who did not complete the PP assessment (see Table 1).

**Table 1:** Sample characteristics at baseline by study arm and completion of the 2-month post-pregnancy (PP) assessment

Characteristic	Total sample (n = 391)	Control (n = 200)	Intervention (n = 191)	t (df = 7); p	Did not complete PP (n = 19)	Completers of PP (n = 372)	t (df = 7); p
Age	27.5 (5.9)	27.7 (6.2)	27.3 (5.7)	0.6; .59	**24.8 (6.8)**	**27.6 (5.9)**	**3.8; .01**
Any secondary education	35.6%	30.5%	41.4%	1.1; .31	31.6%	36.0%	0.8; .46
In a committed relationship	82.3%	84.0%	80.6%	0.9; .39	**63.2%**	**83.3%**	**3.7; .01**
Gestation week	21.3 (22.0)	21.4 (5.6)	21.3 (6.4)	0.2; .87	22.1 (5.8)	21.3 (6.0)	0.8; .44
Months since HIV diagnosis	45.1 (43.8)	46.9 (43.5)	43.2 (44.0)	0.6; .57	**25.6 (29.9)**	**46.1 (44.1)**	**3.2; .02**
Diagnosed with HIV within past 3 mos	20.8%	20.0%	21.5%	0.2; .86	36.8%	19.9%	2.1; .07
Weeks on ART at baseline	172 (177)	179 (176)	164 (179)	0.8; .48	**85 (103)**	**176 (179)**	**5.1; .001**
HIV viral load (log_10_)	1.05 (1.40)	0.96 (1.34)	1.15 (1.45)	0.6; .55	1.57 (2.04)	1.03 (1.35)	1.1; .30
Undetectable HIV viral load	58.3%	60.0%	56.5%	0.4; .70	57.9%	58.3%	0.02; .97
PHQ-9 score	12.7 (5.2)	13.6 (5.6)	11.9 (4.5)	1.3; .23	12.3 (6.5)	12.8 (5.1)	0.2; .85
Depressed (PHQ-9 > 9)	68.3%	72.5%	63.9%	1.0; .35	52.6%	69.1%	0.7; .40

Bolded numbers represent values that are statistically significant (*p* < .05)

### Depression Care

The sample’s mean (SD) PHQ-9 score at baseline was 12.7 (5.2), with 267 (68.3%) having scores (> 9) indicative of clinical depression and warranting treatment; depression levels did not differ between the two study arms at baseline (see Table 1). Only three of the 200 women at the control sites reported receipt of mental health services via usual care processes in the period between baseline and PP assessments, each of whom were treated with antidepressants. Of the 191 women in the intervention arm, 131 initiated depression treatment [84 on PST, 47 on ADT (all but two of whom were treated with fluoxetine)] between baseline and PP assessments, including 117 (95.9%) of the 122 women who screened positive (PHQ-9 > 9) for clinical depression at enrollment, and 10 women who were assessed as being depressed during the monthly ANC routine visits after enrollment; the other 4 women were treated (all with PST) shortly after enrollment, despite having mild depression (PHQ-9 < 10). The amount of time on treatment prior to the PP assessment varied based on the stage of gestation at enrollment and the timing of depression diagnosis. The average time on treatment prior to the PP assessment was 17.2 weeks (median = 16; SD = 8.3), with 120 (91.6%) having at least 8 weeks of treatment, and women on PST had a median of 4 sessions [83.3% had completed the 3 core sessions; mean (SD) number of sessions = 3.8 (1.5)].

Among the 372 who completed the PP assessment, mean (SD) PHQ-9 score at PP was 7.8 (5.6), with 127 (34.1%) having clinical depression; the control group had higher levels of depressive symptomatology (mean PHQ-9 = 10.0 vs. 5.6; t = 8.2, df = 7, p < 0.001), less reduction in depressive symptoms (mean reduction in PHQ-9 = 3.2 vs. 5.7; t = 4.2, df = 7, p < 0.001), and a higher rate of clinical depression (49.7% vs. 18.7%; t = 6.6, df = 7, p < 0.001), compared to the intervention arm. The multivariate logistic regression model showed that the intervention had a strong effect on depression status at the PP assessment, with those in the intervention group being 80% less likely of being clinically depressed compared to women in the control group [Adjusted OR (95% CI) 0.20 (0.05, 0.83); see Table 3]; this result was equivalent to that found in the model with just women who had clinical depression at enrollment (see Table 4).

### PMTCT Adherence and Outcomes

#### Pregnancy Outcome and Post-delivery Processes

In addition to the 372 women who completed the PP assessment, the study coordinators had communication with either the participant or provider of 14 additional cases, resulting in the knowledge of pregnancy outcomes for 386 participants (354 successful live births, 8 still births, 11 neonatal deaths, 2 maternal deaths after delivering the child, and 11 miscarriages/abortions); the remaining five participants were lost to follow-up and categorized as having an unsuccessful pregnancy in the ITT analysis. Among the 354 women who had successful live births, 177 were in each of the intervention and control groups; two of these women had twins, so a total of 356 infants were born from these women. Women in the intervention group were more likely to have their pregnancy result in a successful delivery [92.7% vs. 88.5%; Adjusted OR (95% CI)  1.64 (1.12, 2.42)], and to have their newborn tested for HIV at week 6 [97.8% vs. 96.6%; Adjusted OR (95% CI) 1.63 (1.09, 2.44)], compared to women in the control group. The two groups did not differ on any of the other care processes (see Table 2); 97.2% of the newborns were tested for HIV at six weeks of life, and all tested negative.

**Table 2:** Outcomes at post-pregnancy (PP) assessment in whole sample as well as by study arm (ITT)

	Total sample (n = 391)	Control (n = 200)	Intervention (n = 191)	OR (95% CI) or beta (SE), p
Pregnancy and PMTCT processes				
Successfully delivered child	354 (90.5%)	177 (88.5%)	177 (92.7%)	**1.64 (1.12, 2.42)**
Delivered in health facility^1^	336 (89.6%)	165 (86.8%)	171 (92.4%)	1.85 (0.53, 6.50)
Child given NVP prophylaxis at birth^2^	338 (94.9%)	164 (92.7%)	174 (97.2%)	2.73 (0.45, 16.64)
Child tested for HIV at week 6 of life^2^	346 (97.2%)	171 (96.6%)	175 (97.8%)	**1.63 (1.09, 2.44)**
Child tested HIV-negative at week 6^2^	343 (96.3%)	170 (96.0%)	173 (96.6%)	1.51 (0.96, 2.37)
HIV disease and pregnancy management				
Retained in HIV/ANC care	382 (97.7%)	194 (97.0%)	188 (98.4%)	2.27 (0.43, 11.94)
Mean (SD) number of ANC visits since BL^b^	4.4 (2.0)	4.5 (2.2)	4.3 (1.9)	0.16 (0.44), 0.72
Number of FSG sessions since BL^c^	2.5 (1.9)	2.7 (1.9)	2.2 (1.8)	0.42 (0.32), 0.24
Retained on ART	381 (97.4%)	193 (96.5%)	188 (98.4%)	2.27 (0.43, 11.94)
Mean (SD) ART adherence since BL^3d^	92.9 (18.2)	90.8 (20.2)	95.2 (15.4)	5.33 (5.39), 0.36

Bolded numbers represent values that are statistically significant (*p* < .05)

*BL* baseline

^a^Model includes PHQ-9 score obtained in the screening of eligibility by the nurse as a covariate

^b^Model includes number of ANC visits attended prior to enrollment

^c^Model includes number of FSG sessions attended prior to enrollment

^d^Model includes ART adherence in the six months prior to enrollment; measurement is based on pharmacy refill data

^1^Among the 375 women known to have completed their pregnancy (excludes those with a miscarriage/abortion)

^2^Among the 356 infants successfully delivered (to 354 women, as two women (case 5025 and 8020) had twins)

^3^Among the 381 women on ART at the PP assessment

#### HIV/ANC Care Retention

All but one of the 372 women who completed the PP assessment remained in HIV/ANC care; of the 19 who did not complete the assessment, 10 remained in care (including four who transferred care to another facility and one who withdrew from the study but remained in care). Therefore, a total of 382 (97.7%) remained in ANC care at the PP assessment, and the rate did not differ between the control (97.0%) and intervention (98.4%) group (see Table 2). Between the baseline and PP assessments, the mean number of ANC visits was 4.7 (SD = 1.8; median = 5), and the mean number of FSG sessions attended was 2.5 (SD = 1.9; median = 3); the two study arms did not differ on these measures (see Table 2).

#### ART Use and Adherence

The 382 women who remained in care at the PP assessment, also remained on ART. Pharmacy refill data revealed a mean adherence of 92.9% (SD = 18.2) between baseline and the PP assessment, and there was no difference between the study arms (see Table 2). Most (73.2%) of the sample had good (100%) ART adherence between baseline and PP, and the multivariate logistic regression model showed that the intervention group was marginally more likely to have good ART adherence [Adjusted OR (95% CI) 2.48 (0.84, 7.30); see Table 3]; however, the effect of the intervention was significant in the model that involved only women who were clinically depressed at enrollment [Adjusted OR (95% CI) 3.30 (1.19, 9.19); see Table 4]. In the regression models examining the effects of depression on good ART adherence, depression status at PP was not associated [Adjusted OR (95% CI) 0.86 (0.55, 1.35)], but greater reduction in depressive symptoms from baseline to PP was significantly associated with higher odds of having good ART adherence [Adjusted OR (95% CI) 1.05 (1.01, 1.08)] (see Table 5).

**Table 3:** Regression models examining effects of intervention on depression status, ART adherence, and HIV viral load at post-pregnancy (PP), among the whole sample

Independent variables	Depressed (PHQ-9 > 9) at PP	PHQ-9 at PP	Good (100%) ART adherence from BL to PP	Undetectable HIV viral load at PP	Reduction in HIV viral load from BL to PP
	OR (95% CI)	Beta (SE), p	OR (95% CI)	OR (95% CI)	Beta (SE), p
Main effects					
Intervention	**0.20 (0.05, 0.83)**	**− 4.45 (1.90), 0.05**	2.48 (0.84, 7.30)^T^	1.19 (0.73, 1.93)	0.16 (0.13), 0.27
Covariates					
Depressed (PHQ-9 > 9) at BL	1.85 (0.95, 3.64)		–	–	–
PHQ-9 at BL	–	**0.30 (.09), .01**			
Age	1.02 (0.98, 1.07)	0.03 (0.04), 0.46	0.99 (0.95, 1.03)	1.04 (1.00, 1.07)^T^	0.02 (0.01), 0.06^T^
Any secondary education	1.48 (0.98, 2.24)	0.83 (0.41), 0.08^T^	0.73 (0.39, 1.36)	1.00 (0.72, 1.38)	− 0.06 (0.08), 0.47
In a committed relationship	0.55 (0.27, 1.12)	**− 1.98 (0.69), .02**	1.40 (0.54, 3.62)	0.87 (0.50, 1.52)	− 0.05 (0.10), 0.63
Undetectable HIV viral load at BL	0.99 (0.65, 1.49)	− 0.30 (0.56), .60	0.75 (0.47, 1.21)	**5.07 (3.03, 8.47)**	–
Log_10_ HIV viral load at BL	–	*–*	*–*	–	**0.55 (0.05), < 0.001**
Weeks on ART by PP	1.00 (1.00, 1.00)	− 0.0001 (0.03), 0.27	1.00 (1.00, 1.00)	1.00 (1.00, 1.00)	− 0.0002 (0.0003), 0.59
Weeks between BL and PP	0.99 (0.96, 1.02)	− 0.04 (0.03), .27	0.99 (0.96, 1.01)	1.01 (0.97, 1.06)	0.004 (0.01), 0.70
Number of FSG sessions attended in 6 months prior to PP	0.93 (0.83, 1.04)	− 0.20 (0.13), .16	0.94 (0.80, 1.12)	1.04 (0.86, 1.26)	0.06 (0.05), 0.30

^T^p < 0.10

Bolded numbers represent values that are statistically significant (*p* < .05)

**Table 4:** Regression models examining effects of intervention on depression status, ART adherence, and HIV viral load at post-pregnancy (PP), among the subgroup of depressed (PHQ-9 > 9) women at baseline

Independent Variables	Depressed (PHQ-9 > 9) at PP	PHQ-9 at PP	Good (100%) ART adherence from BL to PP	Undetectable HIV viral load at PP	Reduction in HIV viral load from BL to PP
	OR (95% CI)	Beta (SE), p	OR (95% CI)	OR (95% CI)	Beta (SE), p
Main effects					
Intervention	**0.18 (0.05, 0.69)**	− **5.15 (1.89), 0.03**	**3.30 (1.19, 9.19)**	1.40 (0.75, 2.61)	0.14 (0.14), 0.35
Covariates					
PHQ-9 at BL	–	**0.35 (0.12), 0.02**	–	–	–
Age	1.00 (0.94, 1.08)	− 0.004 (0.05), .95	0.97 (0.91, 1.03)	1.04 (0.99, 1.10)	0.03 (0.01), 0.11
Any secondary education	1.19 (0.70, 2.04)	0.73 (0.56), 0.23	0.74 (0.32, 1.70)	0.93 (0.60, 1.43)	− 0.08 (0.11), 0.51
In a committed relationship	0.60 (0.26, 1.36)	− 1.51 (0.93), 0.15	1.44 (0.57, 3.67)	0.93 (0.37, 2.32)	− 0.03 (0.19), 0.86
Undetectable HIV viral load at BL	1.00 (0.58, 1.71)	0.18 (0.76), 0.82	0.83 (0.43, 1.59)	**5.30 (3.20, 8.77)**	–
HIV viral load at BL (log_10_)	–	–	–	–	**0.57 (0.05), < 0.001**
Weeks on ART by PP	1.00 (1.00, 1.00)	0.0005 (0.002), 0.81	1.00 (1.00, 1.00)	1.00 (1.00, 1.00)	0.0002 (0.0003), 0.47
Weeks between BL and PP	0.99 (0.96, 1.03)	− 0.05 (0.05), 0.30	0.99 (0.96, 1.02)	1.00 (0.96, 1.04)	− 0.007 (0.01), 0.43
Number of FSG sessions attended in 6 months prior to PP	0.92 (0.80, 1.07)	− 0.23 (0.17), 0.22	0.94 (0.79, 1.11)	1.16 (0.93, 1.44)	0.11 (0.06), 0.10

Bolded numbers represent values that are statistically significant (*p* < .05)

**Table 5:** Regression models examining effects of depression status and depression reduction on ART adherence between baseline (BL) and post-pregnancy (PP)

Independent variables	Good ART adherence from BL to PP	
	OR (95% CI)	OR (95% CI)
Depressed (PHQ-9 > 9) at PP	0.86 (.55, 1.35)	**–**
Reduction in PHQ-9 score from BL to PP	–	**1.05 (1.01, 1.08)**
Depressed (PHQ-9 > 9) at BL	0.70 (.41, 1.19)	–
PHQ-9 at BL	–	0.98 (.95, 1.00)^T^
Age	0.99 (.96, 1.03)	1.00 (.96, 1.03)
Any secondary education	0.80 (.39, 1.64)	0.79 (.37, 1.66)
In a committed relationship	1.38 (.59, 3.25)	1.36 (.56, 3.26)
Undetectable HIV viral load at BL	0.74 (.49, 1.12)	0.75 (.51, 1.10)
Weeks on ART by PP	1.00 (1.00, 1.00)	1.00 (1.00, 1.00)
Weeks between BL and PP	0.99 (.95, 1.02)	0.99 (.95, 1.02)
Number of FSG sessions attended in 6 months prior to PP	0.94 (.80, 1.10)	0.94 (.79, 1.12)

^T^p < 0.10

Bolded numbers represent values that are statistically significant (*p* < .05)

#### HIV Viral Suppression

At baseline, the mean log HIV viral load for the sample was 1.05 (SD = 1.40), and 58.3% had an undetectable viral load. At the PP assessment, the sample’s mean log HIV viral load was 0.82 (SD = 1.29), with mean reduction from baseline of 0.23 (SD = 1.34) and 66.2% having an undetectable viral load; the multivariate regression models showed that these did not differ between the intervention and control groups in the whole sample (see Table 3), nor in the models with only women who were clinically depressed at enrollment (see Table 4). Good ART adherence between baseline and PP was also not correlated with undetectable HIV viral load at PP [OR (95% CI) 0.79 (0.52, 1.21)], nor reduction in HIV viral load from baseline to PP [beta (SE) = 0.27 (0.18), t = 1.5, p = 0.18].

## Discussion

This is one of the first studies of the effects of depression care on PMTCT care adherence and outcomes. The M-DEPTH depression care model demonstrated strong effects on alleviating perinatal depression in this sample of women living with HIV. The other pathways in our conceptual model for how depression care would lead to greater maternal viral suppression garnered only modest empirical support, resulting in no evidence of an effect of the intervention on maternal HIV viral suppression at the completion of pregnancy.

Our conceptual framework for how depression care impacts the PMTCT care continuum begins with its most direct pathway, that being its effect on depression. With its reliance on evidence-based depression therapies, the depression care model resulted in a strong alleviation of depression, as expected. Nearly all women who were diagnosed with clinical depression received treatment, either with PST or ADT, and women in the intervention arm were nearly 80% less likely to be clinically depressed at two months post-pregnancy compared to women in the control group. Further detail on the implementation of the two treatment modalities, participants engagement in the treatment process, and predictors of response are available in another publication [44]. It is notable that women in the control group also experienced a reduction in depression between enrollment and the post-pregnancy assessment, which could be attributable to the benefits of usual care, including the FSG program, attention received during the study visits from study coordinators, or social desirability bias that could affect the PHQ-9 scores.

The downstream pathways in our conceptual framework were less supported by our data. We hypothesized that depression reduction would translate to increased ART adherence, and our data did reveal a significant effect; however, the effect was modest and results were mixed. Good ART adherence (i.e., 100% adherence as measured by pharmacy refill data) between baseline and post-pregnancy assessments was associated with the amount of reduction in depressive symptomatology during this time period, but not the presence of clinical depression at post-pregnancy. The subsequent pathway in the model posited that increased ART adherence would be associated with increased HIV viral suppression, but no such relationship was observed in our data. These findings may be largely attributed to the lack of variance in ART use and adherence, which remained generally strong and stable between baseline and post-pregnancy, regardless of depression status.

With the latter pathways of our conceptual model not being evident in our data, it is not surprising that the depression care intervention did not affect ART adherence or HIV care retention, nor HIV viral load at the post-pregnancy assessment. The intervention had a marginal effect on good ART adherence in the whole sample, and a significant effect in the subgroup of women who had clinical depression at enrollment, but this effect was not strong enough to translate into effects on viral load, particularly given the constricted range of ART adherence as described above. Research in Uganda suggests that disengagement from HIV/ANC care tends to increase in the post-partum phase [45]. As more follow-up data is fully collected in this study, we will be able to better assess the full impact of depression care on these HIV care outcomes.

Data related to intervention effects on other PMTCT care processes, namely pregnancy outcome, location of infant delivery, child use of antiretroviral therapy, and child HIV testing and serostatus, did not reveal a meaningful impact of depression care in this sample. We hypothesized that maternal depression may impede these care processes by decreasing the mother’s functioning, motivation, and energy to mobilize herself to visit a health facility, and to follow through with ensuring her child receives important services. Treatment and depression alleviation may help mitigate these harmful effects and enable the mother to closely adhere to these components of PMTCT care. The intent-to-treat analysis found that the intervention group was more likely to have a successful delivery and for the newborn to be tested for HIV shortly after birth, but these differences were due to the larger number of control participants not completing the PP assessment and thus being categorized as having a negative outcome. Like the high level of ART adherence among both depressed and non-depressed women, the lack of meaningful differences on these other care processes reflects the high level of adherence to these care processes in both arms, and regardless of depression status. It is possible that the mother’s motivation to protect her child may help her to adhere to treatment, even when depressed. The study’s later follow-up data will enable us to assess whether adherence to these care processes is sustained in the face of depression, and once the child is at a lower risk for vertical transmission of HIV.

Study limitations include all participants being stable on ART, and over half having undetectable HIV viral load, at enrollment, which may have created a ceiling effect, particularly with regards to ART use and adherence, which were high and stable across the study period in the whole sample. These factors limit the generalizability of our findings. The benefits of depression care would be better evaluated in a sample of women with detectable HIV viral load and lower ART adherence, as well as data further in the post-partum period, when HIV care retention and adherence tend to wane [45]. Also, the measure of ART adherence relied on prescription refill data, the validity of which has support in settings where all patients get their medication from the clinic pharmacy [46]; however, this methodology is low in precision and can overestimate adherence. Further, not all treated women had completed their course of PST or ADT by the time of the post-pregnancy assessment, which may have diluted the intervention effect, but over 90% received at least 8 weeks of treatment.

Using a prospective cohort of pregnant women living with HIV and varying levels of depression, and data available through the completion of pregnancy, our findings revealed strong effects of the M-DEPTH depression care model on depression alleviation, but mixed and modest effect on ART adherence, and no downstream effects on the primary outcome of maternal HIV viral suppression, nor other PMTCT care processes. The sample’s level of viral suppression at enrollment, and relatively uniform high level of ART use and adherence and ANC/HIV care retention from enrollment, impeded the ability to examine the effects of depression alleviation on these outcomes. Further research with a sample more diverse in viral suppression and ART adherence, would help to better evaluate the benefits of depression care for PMTCT care outcomes.

## Data Availability

De-identified dataset and statistical code are available to researchers upon submission of proposal and review by the study team.
